# Quantitative assessment of inter-observer variability in target volume delineation on stereotactic radiotherapy treatment for pituitary adenoma and meningioma near optic tract

**DOI:** 10.1186/1748-717X-6-10

**Published:** 2011-01-27

**Authors:** Hideya Yamazaki, Hiroya Shiomi, Takuji Tsubokura, Naohiro Kodani, Takuya Nishimura, Norihiro Aibe, Hiroki Udono, Manabu Nishikata, Yoshimi Baba, Mikio Ogita, Koichi Yamashita, Tadayuki Kotsuma

**Affiliations:** 1 Department of Radiology, Graduate School of Medical Science, Kyoto Prefectural University of Medicine, 465 Kajiicho Kawaramachi Hirokoji, Kamigyo-ku, Kyoto 602 - 8566 Japan; 2 CyberKnife Center, Soseikai General Hospital,126 Kami-Misu, Shimotoba Fushimi-ku, Kyoto Japan; 3 CyberKnife Center, Tobata Kyoritsu Hospital, Sawami 2 - 5 - 1, Tobata-ku, Kita-Kyusyu, Fukuoka Japan; 4 Toyama Cyberknife Center, Hiyodorijima 1837 - 5, Toyama, Toyama Japan; 5 CyberKnife Center, Okayama Kyokuto Hospital, Kurata 567 - 1, Naka-ku, Okayama, Okayama Japan; 6 Radiotherapy Department, Fujimoto Hayasuzu Hospital, Hayasuzu 17 - 1, Miyakonojo, Miyazaki 885 - 0055, Japan; 7 Tokyo CyberKnife Center, 27 - 1 Negishi, Machida, Tokyo 194 - 0034, Japan; 8 Department of Radiation Oncology, Osaka University Medical School, 2 - 2 Yamadaoka Suita, Osaka Japan

## Abstract

**Background:**

To assess inter-observer variability in delineating target volume and organs at risk in benign tumor adjacent to optic tract as a quality assurance exercise.

**Methods:**

We quantitatively analyzed 21 plans made by 11 clinicians in seven CyberKnife centers. The clinicians were provided with a raw data set (pituitary adenoma and meningioma) including clinical information, and were asked to delineate the lesions and create a treatment plan. Their contouring and plans (10 adenoma and 11 meningioma plans), were then compared. In addition, we estimated the influence of differences in contouring by superimposing the respective contours onto a default plan.

**Results:**

The median planning target volume (PTV) and the ratio of the largest to the smallest contoured volume were 9.22 cm^3 ^(range, 7.17 - 14.3 cm^3^) and 1.99 for pituitary adenoma, and 6.86 cm^3 ^(range 6.05 - 14.6 cm^3^) and 2.41 for meningioma. PTV volume was 10.1 ± 1.74 cm^3 ^for group 1 with a margin of 1 -2 mm around the CTV (n = 3) and 9.28 ± 1.8 cm^3^(p = 0.51) for group 2 with no margin (n = 7) in pituitary adenoma. In meningioma, group 1 showed larger PTV volume (10.1 ± 3.26 cm^3^) than group 2 (6.91 ± 0.7 cm^3^, p = 0.03). All submitted plan keep the irradiated dose to optic tract within the range of 50 Gy (equivalent total doses in 2 Gy fractionation). However, contours superimposed onto the dose distribution of the default plan indicated that an excessive dose 23.64 Gy (up to 268% of the default plan) in pituitary adenoma and 24.84 Gy (131% of the default plan) in meningioma to the optic nerve in the contours from different contouring.

**Conclusion:**

Quality assurance revealed inter-observer variability in contour delineation and their influences on planning for pituitary adenoma and meningioma near optic tract.

## Background

Target delineation is an important issue in radiation oncology, especially for image-guided, high-precision radiotherapy [1]. With increasing conformity of dose delivery, inter-observer variability in tumor identification and delineation plays an ever more critical role, even for uncomplicated lesions [2-6]. Although inter-observer variability in contouring is a well-known fact, we could not find any data on variability in the contouring of benign tumors near the optic tract.

Pituitary adenoma and meningioma are regarded as benign tumors and are rarely treated by radiotherapy if surgery can be performed. However, in cases that are ineligible for surgery due to a risk of excessive surgical complications, radiotherapy can play an important role in treatment for these benign tumors. Radiotherapy was initially performed using conventional technologies (Co-60 or Linac-based units) [7,8]. Stereotactic, single-fraction radiosurgery (SRS) using the Gamma knife was then begun, followed shortly thereafter by stereotactic radiotherapy (SRT) at a number of institutes [9]. The SRT experience for such tumors has been insufficient to develop a consensus on optimal treatment parameters, including prescribed dose and fractionation, especially for hypofractionated SRT. This lack of consensus applies as well to contouring of the planning target volume (PTV).

Therefore, we conducted a multi-institutional study in which participating radiation oncologists delineated tumors and organs at risk (OARs) and created treatment plans using inverse planning software for the CyberKnife System (Accuray, Sunnyvale CA, USA). Participants created treatment plans for two patients, one with pituitary adenoma and second with meningioma. Variability in contouring, planning target volumes, prescribed doses, and doses to OARs was assessed. In addition, we examined the influence of different contouring especially optic tract by superimposing each contour onto the default plan, and we visualized dose distribution using prescribed dose 3-D rendering.

## Methods

Enhanced CT images for Case 1 (pituitary adenoma) and CT and MRI images for Case 2 (meningioma) were obtained at Soseikai General Hospital and sent via internet to seven CyberKnife institutes. For Case 1, CT images were acquired with a SOMATOM Volume Access scanner (Siemens AG, Munchen, Germany) at a 2-mm slice thickness. For Case 2, Contrast enhanced CT images were obtained with a Brilliance CT 64 scanner (Royal Philips Electronics, Euronext: PHIA, Eindhoven, Holland) at a 1.25-mm slice thickness (Default; CT level 35, window 75). MRI images were obtained by an Achieve 1.5 scanner (Royal Philips Electronics, Euronext: PHIA, Eindhoven, Holland) using a 4-mm slice gapless scan (TE 10 ms, TR 450 ms, FA 70°, SPIR). At each CyberKnife institute, the images were transferred to a treatment planning system (TPS; MultiPlan or OnTarget, Accuray) to create a radiotherapy plan for CyberKnife stereotactic radiotherapy (SRT). Participating physicians were required to submit both the printed materials used in their routine clinical work and raw data.

From seven CyberKnife centers, 11 radiation oncologists submitted plans and raw data for the meningioma and 10 for the pituitary adenoma. The collected data contained target volume contours, organs at risk (OARs), and minimum and maximum irradiated dose for those structures. Maximum and minimum doses for the PTV and the maximum dose for OARs were analyzed. Uniformity of dose distribution was assessed in terms of the minimum and maximum prescribed dose for the PTV.

The raw treatment plan data in TPS format were also submitted and analyzed using ShioRIS and ShioRIS-2 (software developed in-house by author H. S.). We superimposed those contours on our default plan (created by author T. T. in Soseikai General Hospital and confirmed by other two physicians), and examined differences in the dose-volume histogram (DVH) for each contour to estimate a prescribed dose for each contoured PTV and organs at risk. The equation; equivalent total doses, EQD2 = n × d × (α/β + d)/(α/β + 2); the dose that would be equivalent to a 2 Gy fractionation was used for the calculation, with EQD2_10_; α/β = 10 for PTV and EQD2_2_; α/β = 2 for OARs. Next, comparison of dose distribution and dose volume renderings for the prescribed dose were analyzed for the pituitary adenoma using ShioRIS-2 in 9 contours.

Generally, treatment plan was made according to the guideline of radiotherapy planning published by Japanese society for therapeutic radiology and oncology 2008 (Table 1)[10,11]. Postsurgical areas are not included intentionally in this trial. However, no consensus was obtained in hypofractionated SRT.

**Table 1:** Reference for planning of pituitary adenoma and meningioma

	Pituitary adenoma	Meningioma
*PTV definitions*		
	PTV = CTV + 1 mm (CTV = GTV),	PTV = CTV + 1 mm (CTV = GTV)
	PTV = CTV + 2 - 4 mm (CTV = GTV)	PTV = CTV + 2 - 3 mm (CTV = GTV)
*Prescribed dose*		
SRS	15 - 20 Gy marginal dose, 25 Gy or more for secreting pituitary adenoma	11 - 18 Gy marginal dose (recommended for 14 Gy or more)
Conventional fractionated SRT		isocenter 45 - 68 Gy/daily 1.8 Gy/fr., D95 50 - 56 Gy/daily 2 Gy/fr.
SRT	45 - 50 Gy/25 - 28 fr.	2 Gy/fr.
		
*Constrains for organs at risk^[11]^*		
SRS	Optic tract < 8 - 10 Gy,	
		
Conventional fractionated		
SRT	Optic tract 50 Gy/25 fr	
		
	Spinal cord < 50 Gy (10 cm or less in length)	
	Retina < 45 Gy	
	Lens < 10 Gy	
	Brain stem < 60 Gy (1/3 volume)	

SRS; stereotactic radiosurgery, SRT; stereotactic radiotherapy,

GTV; gross tumor volume, CTV; clinical treatment volume, PTV; planning target volume, fr.; fraction,

D95; irradiation dose that included 95% volume of PTV

### Case 1. Pituitary adenoma

This patient is a 46-year-old male with pituitary adenoma. He initially presented 4 years before with visual disturbance, and was diagnosed as having a pituitary adenoma. He underwent two surgical interventions, resulting in loss of vision in the right eye; vision was maintained in the left eye. The adenoma gradually progressed, eventually requiring SRT using 25 Gy in 5 equal fractions (5 Gy × 5 times in consecutive 5 days) for a minimum coverage of 90% of the PTV (D90). Default plan used CTV = GTV and PTV = CTV + 1 mm. Conformity index was 1.14. Prescribed doses for OARs are depicted in Table 2.

**Table 2:** Plan characteristics

	Meningioma		EQD2_2_ (Gy)	Pituitary adenoma		EQD2_10_ (Gy)
No of plan	11			10		
Tumor						
Volume of PTV (cm^3^)		8.06 ± 2.45			9.53 ± 1.75	
Prescribed dose	5	30 Gy/5 fr.	40	4	25 Gy/5 fr.	31.3
	1	16 Gy/1 fr.	34.6	2	21 Gy/3 fr.	29.8
	1	21 Gy/3 fr.	29.8	1	22.5 Gy/3 fr.	32.8
	1	24 Gy/5 fr.	29.6	2	24 Gy/5 fr.	29.6
	1	23 Gy/3 fr.	33.9	1	24 Gy/3 fr.	36
	1	24 Gy/3 fr.	36			
						s
Minimal dose/prescribed dose (%)		83.7 ± 9 range 72 - 90			80 ± 12 range 60 - 99	
Maximal dose/prescribed dose (%)		122 ± 15 range 110 - 157			129 ± 17 range 105 - 157	
						
OARs						
(Gy)						
Left eye		0.12 - 2.25			0.02 - 4.48	
Right eye		3.07 - 18.6			0.05 - 4.52	
Brain stem		4.97 - 15.4	4.5 - 19.5		17.3 - 24.74	30.0 - 42.9
Optic chiasm		4.61 - 15.4	4.1 -- 19.9		16.6 - 26.4	22 -- 48
Left lens		0.01 - 2.00			0.03 - 4.08	
Right lens		0.88 - 6.68			0.13 - 3.81	
Left optic nerve		0.79 - 8.96	0.4 - 8.5		12.4 - 23.4	23 - 50
Right optic nerve		7.93 -- 26.4	14.6 - 47.9		0.37 - 23.4	17.5 -- 57.5

EQD2 = n × d × (α/β+d)/(α/β+2), EQD2_10_; α/β = 10 for PTV and EQD2_2_; α/β = 2 for organs at risk

OARs; organs at risk, fr.; fraction

### Case 2. Meningioma

This patient is a 50-year-old female with sphenoid ridge meningioma. She experienced back pain while performing nursing care for her mother three years before, and was diagnosed at the time as having a meningioma. During several years of follow-up the tumor grew, eventually requiring surgical intervention. Thereafter, a residual tumor grew slowly and she was recommended for further treatment with the CyberKnife. She received SRT using 30 Gy in 5 fractions (6 Gy × 5 times in consecutive 5 days) for D90. Default plan used CTV = GTV and PTV = CTV + 1 mm. Conformity index was 1.12. Those plans were verified by other two physicians, and used as a control references.

### Statistical Analysis

All statistical analyses were carried out with the Statview-v5.0 software program. Student's t-test was used for normally distributed data and the Mann Whitney U-test for skewed data. Percentages were analyzed with the Chi-square test. A value of *p *< 0.05 was considered to be statistically significant.

## Results

### Case 1. pituitary adenoma

**Figure 1:**
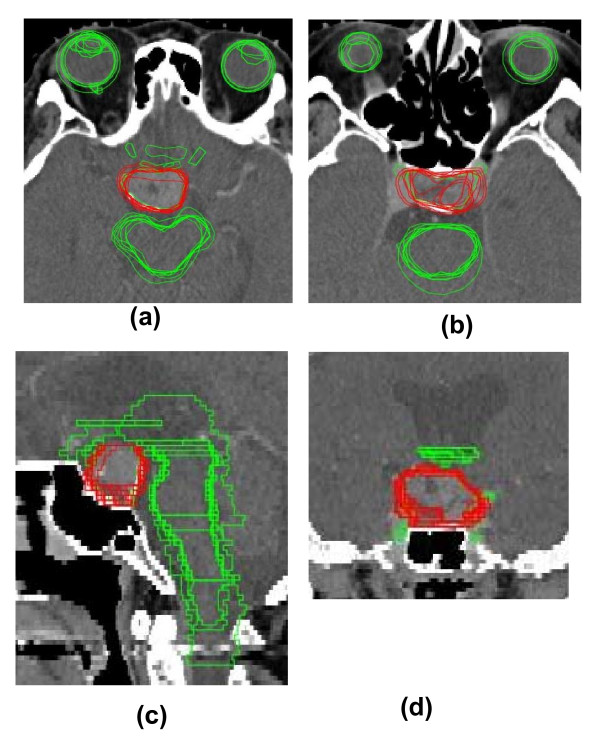
**Contours superimposed on the default CT image (Pituitary adenoma)**. (a) (b) axial section (c) sagittal section (d) coronal section.

**Figure 2:**
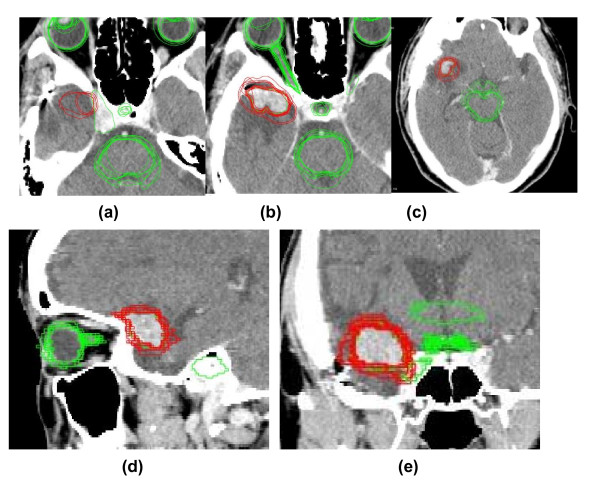
**Contours superimposed on the default CT image (Meningioma)**. (a) (b) (c) axial section (d) sagittal section (e) coronal section The red lines depict planning target volumes, and the green lines depict delineations of organs at risk from each clinician.

**Figure 3:**
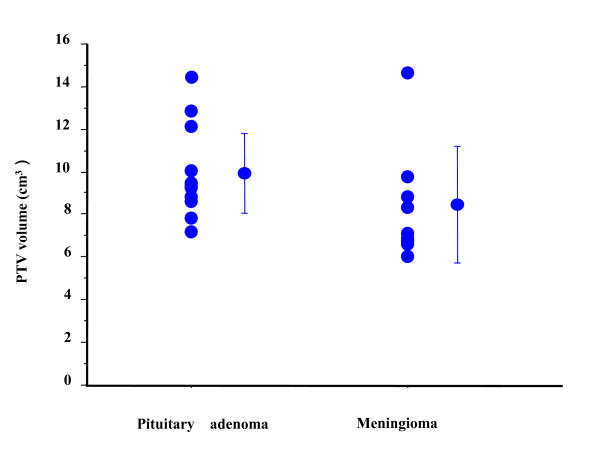
Variation in planning treatment volume (PTV) in pituitary adenoma and meningioma cases.

Each contour was superimposed on the original CT images (Figure 1). Three physicians used PTV = CTV + 1 mm (group 1 with a margin of 1 mm around the CTV). Seven used a protocol in which the PTV = CTV = GTV (group 2 with no margin). The median PTV was 9.22 cm^3 ^(range, 7.17 - 14.3 cm^3^; Figure 2); the ratio of the largest to the smallest contoured volume was 1.99. Group 1 used PTV volume 10.1 ± 1.74 cm^3 ^and group 2 used 9.28 ± 1.8 cm^3 ^(p = 0.51, n.s.). Four physicians used D90 (9.37 ± 0.4 cm^3^) and six used D95 (9.7 ± 2.6 cm^3^, n.s. vs. D90 group) as dose prescription methods. The average of the smallest prescribed dose divided by the prescribed dose in the PTV was 80% and the mean maximum dose was 129%. The irradiated doses for the OARs are depicted in Table 2. The irradiated dose to the intact left optic nerve was kept below 50 Gy (EQD2_2_) according to guideline [10,11], whereas the right optic nerve in which vision was already lost received 57.5 Gy (EQD2_2_). Thus, no plan exceeded the critical dose for the OARs [10,11]. Next, we analyzed DVHs by superimposing the dispatched contours onto the original default plan (25 Gy in 5 fractions for D90; Figure 3). The prescribed dose for D90 varied from 23.34 - 24.78 Gy (median: 24.68 Gy). Maximum dose for left optic nerve ranged from 8.78 - 23.64 Gy (median: 12.41 Gy). Although the default plan delivered a maximum dose of 8.79 Gy to the left optic nerve, the maximum dose was increased up to 23.64 Gy (268% higher dose than the default plan) in the comparison contours. Therefore, contour deviation could cause an unintended higher dose delivered to the OARs.

### Case 2. meningioma

**Figure 4:**
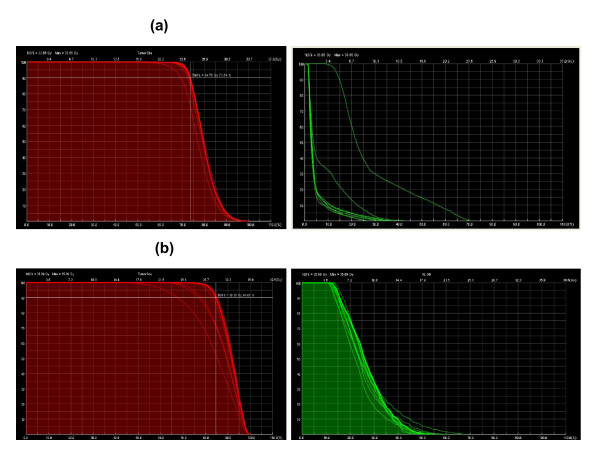
**Influence of different contours on the DVH analysis**. Each contour was layered over the original default plan (Soseikai General Hospital). The dose calculation was made by ShioRIS, and the DVH was calculated using ShioRIS-2. a) Pituitary adenoma:10 contours Left panel. PTV. Default D90 = 25 Gy/5 fractions. According to the applied contours, the D90 median dose was 24.68 Gy (23.34 - 24.78 Gy). Right panel. OAR (left optic nerve). Left optic nerve received 8.79 Gy in default plan (made by T. T.), median 12.41 Gy (8.78 - 23.64 Gy; 23.64 Gy = 268% of default plan) b) Meningioma: 11 contours Left panel. PTV. Default D90 = 30 Gy/5 fractions. According to the applied contours, the D90 median dose was 30.29 Gy (24.24 - 30.66 Gy). Right panel. OAR (right optic nerve). The right optic nerve received a median dose of 19.39 Gy (16.21 - 24.84 Gy; 18.9 Gy in the default plan). Therefore, some plans used 24.24 Gy as a D90 prescribed dose (19% lower dose than anticipated in widen PTV group) when using their contoured PTV. In addition, a higher maximum dose of 24.84 Gy (131% higher dose than default plan 18.9 Gy) was delivered to the right optic nerve in contours used in some institutes.

Each contour was superimposed on the original CT images (Figure 2). Two physicians used PTV = CTV + 1 mm, and one used PTV = CTV + 1 - 2 mm (group 1 with a margin of 1 mm around the CTV). Seven used protocol PTV = CTV = GTV (group 2 with no margin). The median PTV was 6.86 cm^3 ^(range, 6.04 - 14.6 cm^3^) (Figure 3), and the ratio of the largest to the smallest contoured volume was 2.41. Group 1 used larger PTV volume (10.1 ± 3.26 cm^3^) than group 2 (6.91 ± 0.7 cm^3^, p = 0.03). Four physicians used D90 (6.7 ± 0.4 cm^3^), and seven used D95 (8.1 ± 1.2 cm^3^, vs. D90 group) as a prescribed dose. The average minimum prescribed dose (%) in the PTV was 83.7% and the mean maximum dose was 122%. The prescribed dose for OARs was assessed in 11 cases and is depicted in Table 2. No plan exceeded the determined critical dose for OARs [10,11]. Next, the DVHs were reanalyzed by superimposing the contours from participants onto the default plan (Figure 4). The median value for the D90 prescribed dose for PTV was 30.29 Gy (24.24 - 30.66 Gy), and the maximum dose received by the right optic nerve had a median value of 19.39 Gy (16.21 - 24.84 Gy). Therefore, some plans used 24.24 Gy as a D90 prescribed dose (19% lower dose than anticipated) when using their contoured PTV. In addition, a higher maximum dose of 24.84 Gy (131% of the default plan dose of 18.9 Gy) was delivered for the right optic nerve in the contours used in some institutes.

## Discussion

Inter-observer variation is a well-known problem in medical practice. Gardenia et al. first reported on this issue in the 1950 s [3], and it became a subject for discussion in the radiotherapeutic community in the 1970 s. In the 1990 s, many articles were published about inter-observer variation for a variety of cancers: prostate cancer [4], brain tumors [5], breast cancer [6] head and neck cancer [12,13], and lung cancer [14,15]. However, we were unable to find any papers that examined inter-observer variation for pituitary adenoma and meningioma; to the best of our knowledge, this is the first such report.

DVHs analysis by superimposing different contours from multiple clinicians onto the default treatment plan showed higher maximal dose for optic tract (Figure 3). It was increased to 23.64 Gy (268% higher dose than default plan) for the pituitary adenoma and 19.39 Gy (131%) for the meningioma. These results imply that contour deviations across plans could easily cause unexpectedly higher doses to OARs. On the other hand, some comparison plans prescribed 19% lower does than the default 24.24 Gy in the meningioma. Although the dose to PTV is not a matter of this study because it will be changed by physician's decision (PTV definition etc.), we can suggest that there are such a variety of different SRT plans using same CT images.

Several limitations should be considered in our study. At first, BED assessment is not validated in hypofractionated SRT, however it is an only method to compare different fractionation quantitatively at present. Next, although we used default plan as a control references after confirmed by other two physicians, there is neither consensus in contouring nor planning in these area, so that in fact it is only simulation examination. Thirdly, although we confirmed precision of fusion software by visual inspection at least by other two physicians, accuracy of fusion is still qualified by subjective methods.

To obtain reproducible outcomes using an inverse plan, consensus among the participants should be reached in advance to avoid uncertainty; for example, definitions of major violations should be provided and training sessions made available for participants to improve the conformity of their plans to an agreed upon benchmark. These results underline the importance of QA assessment for reproducible outcomes, not only in contouring and the setting of dose constraints, but also for planned dose distributions especially in a multi-clinician study. We should keep in mind the risk of such new techniques as cyberknife if the QA is not followed.

In conclusion, quality assurance revealed inter-observer variability in contour delineation of pituitary adenoma and meningioma near optic tract.

## Competing interests

The authors declare that they have no competing interests

## Authors' contributions

HY conceived of this study and drafted manuscript, HS made software and participated in the design of this study. TT, NK and TN participated in confirmation of default plan and the statistical analysis. HY, HS, TT, NK, TN, NA, HU, MN, YB, MO, KY and TK made plan and participated coordination and helped to draft the manuscript. All authors read and approved the final manuscript.
